# Efficacy and Safety of Sildenafil in Combination with Dapagliflozin Versus Dapagliflozin Monotherapy in the Management of Heart Failure with Pulmonary Arterial Hypertension: A Randomized Controlled Trial

**DOI:** 10.3390/ph18111663

**Published:** 2025-11-03

**Authors:** Esraa M. Abdallah, Marwa Kamal, Khaled Elkhashab, Mahmoud A. Mohamed, Ammena Y. Binsaleh, Marwa Mohsen, Raghda R. S. Hussein

**Affiliations:** 1 Department of Clinical Pharmacy, Faculty of Pharmacy, Fayoum University, Fayoum 63514, Egypt; ema17@fayoum.edu.eg (E.M.A.); mka05@fayoum.edu.eghussein5 (M.K.); 2 Department of Cardiology, Faculty of Medicine, Fayoum University, Fayoum 63514, Egypt; khashabkhaled6@gmail.com; 3 Hikma Pharmaceutical Company, Beni-Suef 62521, Egypt; mmabdelfatah@hikma.com; 4 Department of Pharmacy Practice, College of Pharmacy, Princess Nourah bint Abdulrahman University, P.O. Box 84428, Riyadh 11671, Saudi Arabia; aysaleh@pnu.edu.sa; 5 Department of Clinical Pharmacy, Faculty of Pharmacy, Beni-Suef University, Beni-Suef 63511, Egypt; marwa.mohsen@pharm.bsu.edu.eg

**Keywords:** heart failure, secondary pulmonary hypertension, dapagliflozin, sildenafil

## Abstract

**Background**: Heart failure (HF) with pulmonary hypertension due to left-sided heart disease (PH-LHD) is associated with poor prognosis. Dapagliflozin showed benefits in terms of ejection fraction (EF); meanwhile, sildenafil improved pulmonary pressures and right ventricular function in PH -LHD in recent clinical studies. This study assesses the potential additive effects of dapagliflozin and sildenafil on cardiac function and pulmonary hemodynamics in this population. **Methods**: In this prospective, randomized, controlled trial, 93 participating patients with HF and PH-LHD were randomly assigned to receive dapagliflozin (control group, *n* = 48) or dapagliflozin plus sildenafil 25 mg/day (test group, *n* = 45) in addition to conventional therapy for HF for 12 weeks. The primary outcomes were assessing changes in echocardiographic hemodynamic parameters. Secondary outcomes included outcomes, changes in cardiac enzyme (troponin), kidney function (serum creatinine), and lipid profile. **Results**: The average baseline median left ventricular ejection fraction (LVEF) for both groups was 30%, and the Pulmonary Artery Systolic Pressure (PASP) median was 50 mmHg. At follow-up, PASP had declined, and EF had improved compared to baseline. However, there were no statistically noticeable variations between the groups (*p* = 0.458, 0.331, respectively). No notable changes were observed in secondary and safety outcomes, including hospitalization rate, number of deaths, kidney function, and cardiac enzymes (*p* = 0.524, 1, 0.923, and 0.574, respectively). **Conclusions:** Addition of sildenafil to dapagliflozin did not demonstrate any significant clinical or hemodynamic benefit over dapagliflozin monotherapy in HF patients and PH-LHD. Further studies are warranted to evaluate the effects over the long term.

## 1. Introduction

A clinical condition known as heart failure (HF) is characterized by warning signs and/or symptoms that are associated with abnormalities in heart structure or function, and which are confirmed by elevated levels of natriuretic peptides and/or signs of pulmonary or systemic congestion [1]. Three distinct phenotypes were formed from the stratification of HF patients according to the proportion of blood expelled by the left-sided ventricle per heartbeat, known as left-sided ventricular ejection fraction (LVEF). HF with decreased ejection fraction (HFrEF), which is indicated by an LVEF of 40% or less; HF with mildly reduced ejection fraction (HFmrEF), which is defined as an LVEF ranging from 41% to 49%; and finally, HF with preserved ejection fractions (HFpEF), which is indicated by an LVEF of 50% or higher [2]. HF is a multifaceted, potentially fatal condition that impairs heart function and is associated with significant health complications, a decline in functional ability, and lower life quality. Furthermore, HF can result in considerable healthcare costs, which compromise a global health issue [3]. Consequently, lessening the social and economic impacts of HF has become a crucial global public health issue [4]. Because of factors including aging populations, increased post-myocardial infarction survival, and advancements in HF care and outcomes, the prevalence of HF is continuously increasing even though it has leveled out globally and even decreased in wealthy nations [5].

Pulmonary arterial hypertension (PH) is a critical, progressive, and potentially fatal pulmonary disease [6]. It is related to elevated pressure in the pulmonary arteries, structural alteration of the pulmonary blood vessels, and increased pressure in the pulmonary arteries. These pathological alterations raise the right ventricle’s afterload, which typically results in maladaptive structural remodeling of the right ventricle and ultimately causes death [7]. PH on echocardiography was defined as having a pulmonary artery systolic pressure (PASP) higher than 36 mmHg [8].

Based on the underlying etiology, PH is divided into five types: Group I is caused by PH. Group 2 includes patients with left-sided heart disease, while Group 3 includes patients with lung disease that has been present for a long time. A pulmonary arterial obstruction causes Group 4, while pulmonary hypertension with unclear or multifactorial origins causes Group 5 [9]. PH resulting from left-sided heart disease, which is known as Group 2 PH (PH-LHD), represents the most common and prevalent form of pulmonary hypertension. This condition is characterized by chronic elevation of the left-sided filling pressures transmitted to the pulmonary circulation, frequently leading to a challenging prognosis [10].

First, beta-blockers are used to treat HFrEF. Second, mineralocorticoid receptor antagonists (MRAs) are used. Third, angiotensin-converting enzyme inhibitors (ACEIs), angiotensin receptor-neprilysin inhibitors (ARNIs; sacubitril/valsartan), or angiotensin receptor blockers (ARBs) are used, and fourth, sodium–glucose cotransporter 2 (SGLT2) inhibitors [11].

Dapagliflozin, which is a sodium–glucose cotransporter 2 inhibitors (SGLT2i), represents a new class of antidiabetic drugs that act on the proximal renal tubules to inhibit glucose reabsorption, promoting glycosuria and thereby improving glycemic control [12]. Multiple major clinical trials, such as EMPA-REG OUTCOME, CANVAS, DECLARE-TIMI 58, and CREDENCE, have demonstrated that SGLT2 inhibitors can enhance both cardiovascular and renal clinical outcomes [13]. Additionally, they have demonstrated significant cardiovascular advantages in individuals with acute or chronic HF, as well as in those with or without diabetes [14]. These advantageous effects seem to be mediated by a notable reduction in intracellular sodium, a mechanism that is recognized for its cardioprotective effects by preventing oxidative stress and the subsequent death of cardiomyocytes [15]. This may lead to reduced hospitalization rates for HF, improved kidney function, and reduced cardiovascular mortality [16].

The 2021 European Society of Cardiology (ESC) guidelines recommend two SGLT2 inhibitors, dapagliflozin (DAPA) and empagliflozin (EMPA), as first-line therapy for management of patients having HFrEF, alongside other guideline-directed first-line agents, irrespective of diabetes status [17]. According to the 2023 ESC guideline update, the administration of SGLT2 inhibitors (class IA) is also indicated for individuals with HFpEF and HFmrEF. Consequently, SGLT2 inhibitors are currently the only therapy indicated for all HF patients to improve prognosis, irrespective of LVEF [18].

Sildenafil is a selective Phosphodiesterase Type 5 (PDE-5) inhibitor. These agents received U.S. Food and Drug Administration (FDA) approval in 2005 for treatment of PH and have shown promise in improving quality of life [19].

The rationale for using PDE-5 inhibitors in the treatment of PH depends on their ability to enhance nitric oxide-related signaling mechanisms by reducing the hydrolysis of cyclic guanosine monophosphate (cGMP). By increasing cGMP levels, these agents promote vasodilation, exert both antiproliferative properties and pro-apoptotic effects, and may help reverse pulmonary vascular remodeling [20]. Evidence also suggests that PDE-5 inhibitors could directly enhance the right ventricle’s contractility by increasing cyclic adenosine monophosphate (cAMP) levels [21]. Research on patients diagnosed with mixed pre- and post-capillary pulmonary hypertension (Cpc-PH) and HFpEF has shown that PDE-5 inhibitors demonstrated long-term efficacy by reducing PH and improving right ventricular contractility [22]. These hemodynamic and functional effects may be clinically relevant, but additional studies are required to clarify the precise role of PDE-5 inhibitors [23].

Despite advancements in HF therapy, management of patients with PH remains a clinical challenge. While medications like (sacubitril/valsartan) and dapagliflozin have shown individual benefits in improving cardiac remodeling, symptoms, and outcomes in HF patients [24,25], and sildenafil has demonstrated a reduction in PH and improved right ventricular function among individuals with PH-LHD, the combined effects of dapagliflozin and sildenafil have not been systematically studied.

Our study aims to explore the potential synergistic effect of sildenafil combined to dapagliflozin in the treatment of HF patients complicated by PH-LHD and compare the results with dapagliflozin monotherapy.

Because dapagliflozin and sildenafil have complementary modes of action that target different aspects of HF and PH, their combination seems rational. Through glucosuria, natriuresis, and osmotic diuresis, dapagliflozin reduces cardiac preload and plasma volume while conferring cardiovascular and renal protection. SGLT2 inhibitors (as dapagliflozin) are currently the only therapy indicated for all HF patients to improve prognosis, irrespective of LVEF; it also can reduce cardiovascular events and mortality in HF patients [18,26]. In contrast, sildenafil primarily functions as a pulmonary vasodilator by improving right ventricular function, reducing pulmonary vascular resistance, and boosting nitric oxide–cGMP signaling [20,21]. When used together, the potential exists for additive benefits, with dapagliflozin addressing systemic congestion and metabolic burden and sildenafil alleviating pulmonary vascular load. However, safety considerations remain critical, as combined therapy may increase the risk of hypotension, renal dysfunction, or overlapping adverse effects, and clinical data are still limited, warranting further trials before routine use can be recommended.

## 2. Results

In this study, 45 of the 93 patients were assigned to the test group and 48 to the control group; a summary of their baseline characteristics is included in Table 1. The patients’ median age was 54 years and 37% were female. The average baseline median LVEF was 30 (27–40) %, whereas the PASP baseline median was 50 (45–53) mmHg.

### 2.1. Outcome Measures

#### 2.1.1. Effect on Echocardiographic Hemodynamic Parameters

**Figure 1:**
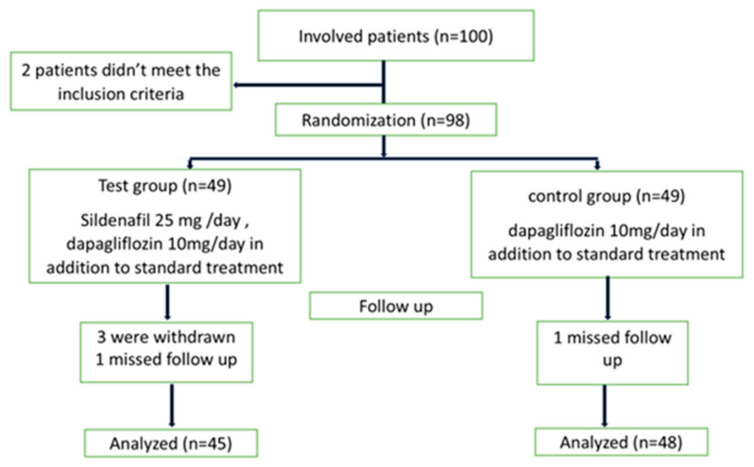
Patient disposition.

**Figure 2:**
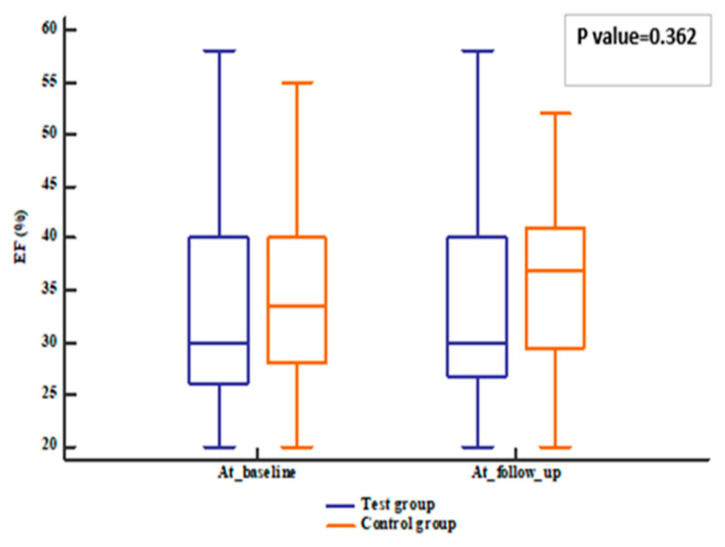
EF of the studied groups. EF: ejection fraction.

**Figure 3:**
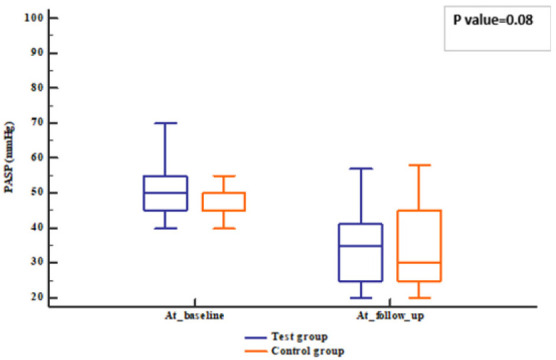
PASP of the studied groups. PASP: Pulmonary artery systolic pressure.

**Figure 4:**
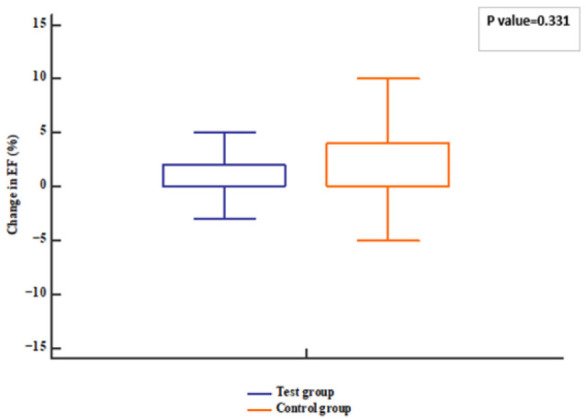
Change in EF of the studied groups. EF: ejection fraction.

**Figure 5:**
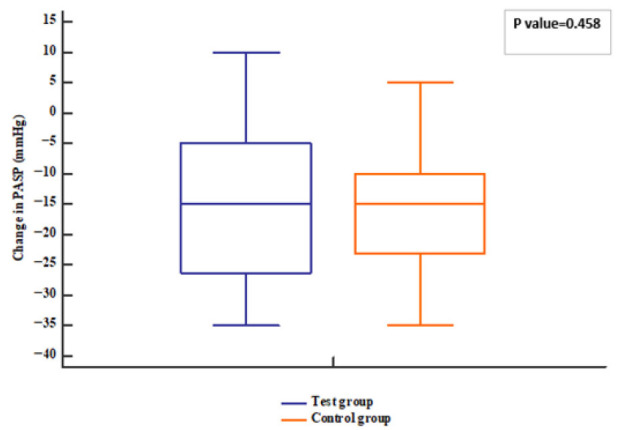
Change in PASP of the studied groups. PASP: Pulmonary artery systolic pressure.

The patients’ follow-up was represented in Figure 1. Regarding the echocardiographic hemodynamic parameters, PASP tended to decline in both groups (*p* value < 0.001) after 12 weeks; meanwhile, EF increased compared to baseline (*p* value = 0.006). However, no differences were observed between groups (*p* value = 0.331 for EF and 0.458 for PASP) (Figure 2, Figure 3, Figure 4 and Figure 5).

#### 2.1.2. Effect on Cardiac Enzyme, Kidney Function Parameters and Lipid Profile

No significant differences in kidney function (creatinine (mg/dL)) and cardiac enzyme (troponin (ng/mL)) were observed (*p* value = 0.923 for creatinine and 0.574 for troponin) (Table 2). Changes in lipid profile were similar in both groups; cholesterol and LDL in the test group and control group were significantly decreased after 12 weeks of follow-up (*p* value < 0.05). HDL in the test group was higher at follow-up, and in the control group, it was insignificantly different between baseline and follow-up, without a significant difference in either group (*p* value = 0.152). Triglycerides in the test group and control group were insignificantly different at follow-up (*p* value = 0.598) (Table 2).

### 2.2. Safety Outcomes and Adverse Events

The differences in safety outcomes were not statistically significant between the two groups. These outcomes included adverse events, cardiovascular death, and worsening HF events. The proportion of patients hospitalized was 6.66% for the test group and 8.33% for the control group (Table 2), and two deaths from worsening heart failure occurred in each group over the 12-week follow-up, resulting in mortality rates of approximately 4.44% in the test group and 4.17% in the control group (*p* value = 1) (Table 3).

No severe adverse effects were reported throughout the study period, and both interventions were generally well tolerated. No patient needed to discontinue taking the study medication permanently because of hypotension or any other side effect.

## 3. Discussion

SGLT2 inhibitors, such as dapagliflozin, are considered a substantial addition to HF treatment, irrespective of LVEF, diabetic status, or the acuity of the clinical setting, and appear to be effective and well tolerated in most clinical HF scenarios [27]. Our study is, to our knowledge, the first randomized controlled trial to specifically evaluate the incremental efficacy of adding sildenafil to a baseline regimen that already includes SGLT2 inhibitor therapy in patients diagnosed with HF and evidence of PH-LHD without needing an invasive test compared to dapagliflozin monotherapy. Most existing studies were conducted before the widespread adoption of SGLT2 inhibitor-based therapy, and therefore, their findings may not accurately reflect the current therapeutic landscape. Given the known pulmonary vascular and diuretic effects of both agents, we hypothesized a potential additive benefit in patients with HF and PH-LHD. In this trial, no beneficial clinical or hemodynamic effects were detected from the addition of sildenafil to dapagliflozin therapy in these patients. According to these findings, dapagliflozin plus optimized HF therapy may provide clinical benefits to both groups; however, the incremental value of sildenafil in this population remains uncertain.

Other research has found that administering PDE-5 inhibitors to HF patients, and complicated by PH-LHD, produced neutral or moderate results, which aligns with our findings. This consistency clearly suggests that the therapeutic window for additional incremental improvement from pulmonary vasodilators may be effectively narrowed by the robust benefits provided by maximal Guideline-Directed Medical Therapy (GDMT). For example, a larger clinical trial conducted by Redfield and co-workers, which enrolled patients diagnosed with HFpEF both with and without PH, demonstrated that sildenafil had no beneficial therapeutic effects on symptomatic status, functional exercise capacity, and pulmonary hemodynamic parameters [28]. Likewise, Hoendermis and colleagues evaluated the use of sildenafil in individuals who have HFpEF and predominantly post-capillary PH and showed no significant symptomatic improvement, physical performance, or hemodynamic parameters obtained through invasive methods after 12 weeks of treatment [29]. Moreover, a study by Cooper and colleagues concluded that in patients with HFrEF and PH, sildenafil did not positively affect symptoms, health-related quality of life, or capacity of functional exercise [30].

Findings of another recent study which supported the efficacy of pulmonary vasodilatation—targeted therapy for management of patients with PH-LHD—are controversial [31]; they explained that, as its benefit may be restricted by the injured left ventricle’s inability to hold the extra volume of blood, the pressure-relieved right-sided ventricle delivers without raising left-sided filling pressures even further. A systematic review and meta-analysis published recently has also highlighted the heterogeneous response to sildenafil in PH-LHD, supporting the need for individualized therapy [32]. Notably, ten patients in the test group and eight in the control group were initiated on sacubitril/valsartan during the follow-up period in our study. The initiation of this high-potency GDMT agent serves as critical supporting evidence for our interpretation. This background therapy may have played a role in the observed improvements in clinical outcomes, such as a reduction in hospitalizations, decrease in mortality, improvement in symptoms (e.g., NYHA class), and EF [33,34], potentially enhancing the effect of dapagliflozin; as such, this observation strongly suggests that any potential effect from sildenafil was masked or outweighed by the robust benefits of modern HF treatment, further emphasizing the limited incremental benefit of pulmonary vasodilators in HF patients having PH-LHD and already receiving optimized therapy.

On the other hand, our results are in significant contrast to a number of small-scale clinical trials that indicate that short-term PDE-5 inhibitor medication may result in general improvements in exercise capacity, symptoms, and life quality [35,36]. These trials reported that sildenafil has been shown to improve pulmonary hemodynamics when taken chronically, suggesting that it may help slow or perhaps reverse the pathological vascular remodeling associated with PH.

Lewis and colleagues examined the effects of sildenafil being administered to 34 people with a confirmed diagnosis of HFrEF and PH at doses of 25–75 mg three times a day for 12 weeks. They found that sildenafil enhanced patients’ life quality and improved exercise capacity, as demonstrated by higher maximum consumption of oxygen (VO_2_) and 6 min walk test (6MWT) distance; patients on sildenafil also had lower rates of HF hospitalizations. However, PH, as determined by right cardiac catheterization, showed no significant changes [37]. In a longer-term study, Guazzi and co-workers evaluated the effects of sildenafil over a six-month period, involving 46 patients with HFrEF and PH; treatment resulted in improvements in brachial artery flow-mediated dilatation (FMD), decreases in PASP, and increased exercise tolerance and breathlessness, which was confirmed by improved peak VO_2_ [36]. Likewise, in a randomized, placebo-controlled clinical trial including 19 patients with HFrEF, Behling and colleagues found that sildenafil markedly increased exercise capacity and decreased echocardiographically estimated PASP [35]. This discrepancy is likely multifactorial. Notably, all of the previous studies used higher doses of sildenafil (e.g., 25–75 mg three times daily) compared to our study (25 mg/day); however, none of them reported the use of SGLT2 inhibitors such as dapagliflozin as part of baseline therapy. Given the known pulmonary vascular and diuretic effects of both agents [38,39], the use of a lower dose of sildenafil in our study may have contributed to the absence of significant hypotensive events among enrolled patients.

As reported in a study by Cooper and Lewis [30,37], despite being inconsistent with that of Behling [35] and Guazzi [36], we observed that PASP was not significantly reduced following treatment with sildenafil. This discrepancy might be attributed to variations in patient inclusion criteria. This clinical trial did not exclude patients with permanent atrial fibrillation (AF), a population at risk for chronically elevated left atrial pressures. In contrast, many earlier studies assessing sildenafil in HFrEF populations did not include patients with AF [35,36,37,40]. It is possible that the constriction of pulmonary arterioles has become a permanent feature of pulmonary arteriolar remodeling. The findings in our patients suggest that the window of opportunity for PH reversal may have already been missed.

Despite death in both arms, the rates were statistically insignificant and nearly identical. Over the duration of the 12-week follow-up, two deaths happened in each group. This mortality rate, while remarkable, aligns with the expectations of populations with advanced HF, particularly those with pulmonary hypertension. The observation underscores how serious this condition is and the importance of ongoing therapeutic innovation. As both treatments were generally well tolerated and no serious side effects were recorded during the trial period, it appears that combination therapy at these dosages is both feasible and safe for short-term usage in this patient population.

### Limitations and Future Work

This study’s limitations include a small sample size, a single-center design, and a short follow-up period (12 weeks), which limit our capacity to assess and validate long-term clinical outcomes including significant cardiovascular events and sustained ventricular remodeling. However, even small-scale trials and safety data collected during this initial 12-week period can provide useful guidance by assessing the strength and variety of treatment effects, which can guide the design and sample size calculations for subsequent definitive trials. Additionally, our sample did not contain patients with severe PH (defined as PASP >100 mmHg); therefore, it may have excluded those who may benefit greatly from pulmonary vasodilation. Large-scale, multicenter trials with longer follow-up periods and more rigorous hemodynamic evaluations are necessary in the future. In our study, right heart catheterization was not required for enrollment; instead, we relied on non-invasive Doppler echocardiography to assess pulmonary hypertension. Although non-invasive and invasive measurements generally correlate well, this non-invasive approach limited our ability to assess the PH’s reversibility or to differentiate between isolated pre-capillary pulmonary hypertension (ipcPH) and (cpcPH). Gradual titration of sildenafil under close blood pressure monitoring would be more helpful to better define the potential therapeutic role of this combination, instead of the steady low dose used in this trial.

## 4. Patients and Methods

### 4.1. Design, Setting, and Participants

Our study is a randomized controlled trial in which approximately 100 patients were recruited, with patients’ disposition shown in Figure 1. This was an exploratory, single-center pilot study conducted from 20 January 2025 to 1 August 2025, and patients were collected from Fayoum University Hospital and its associated outpatient clinics in Egypt. The Research Ethics Committee of the Faculty of Medicine, Fayoum University, Fayoum, Egypt (NO: RHDIRB-NA-101023-01UC-GU-No.1023) reviewed and approved the study protocol. Written informed consent was provided from all patients who participated in the study. The trial was registered with ClinicalTrials.gov (ID: NCT06778330). Of 100 patients, 93 completed the study having HF, including patients with [HFrEF], [HFmrEF], and HFpEF up to ≤60%, and New York Heart Association [NYHA] (functional class II–III), complicated by PH-LHD, characterized by a PASP of 40 mmHg or higher. Diagnosis was based on echocardiographic findings and clinical symptoms, including exertional dyspnea and reduced exercise tolerance. Some patients also experienced chest discomfort, syncope, or peripheral edema [41]. The enrolled patients had no documented episodes of hypotension; systolic blood pressure average was typically in the range of 120–130 mmHg, and diastolic pressure was between 70 and 80 mmHg. Patients were excluded from the study if they had a history of severe allergic reactions or hypersensitivity to sildenafil, dapagliflozin, or related medications; patients with contraindications to the use of sildenafil or dapagliflozin, such as advanced decline in renal function (e.g., estimated glomerular filtration rate < 30 mL/min/1.73 m^2^ for dapagliflozin) [42,43]; and those with progressive liver dysfunction (e.g., Child–Pugh class C) or active liver disease [44]. Additional exclusion criteria were patients aged less than 18 years, significant cardiac valvular disorders, and patients suffering from PH resulting from conditions other than HF (e.g., idiopathic PH, pulmonary veno-occlusive disease, and hemangiomatosis of the pulmonary capillaries). Pregnant and breastfeeding women as well as patients with a predicted life expectancy of less than six months were also excluded.

### 4.2. Interventions

Patients were randomly assigned to two equal groups. The test group received sildenafil (25 mg/day), in addition to standard treatment for HF; the control group received dapagliflozin (10 mg/day) as a part of standard treatment for HF [45,46]. Patients were subsequently followed up for 12 weeks.

The four cornerstone standard therapies for HF include: first, beta-blockers; second, MRAs; third, ACEis, (ARNI; sacubitril/valsartan) or ARBs; and fourth, (SGLT2) inhibitors.

### 4.3. Main Outcomes and Measures

The primary outcome was to assess the short-term efficacy of the combination therapy by measuring the changes in the echocardiographic hemodynamic parameters, including LVEF and PASP. Secondary and safety outcomes were designed to evaluate the overall tolerability and safety of the combined regimen within this initial 12-week window. These outcomes included the incidence of adverse events (AEs), cardiovascular death, or worsening HF events leading to hospitalization, alongside changes in cardiac enzyme (troponin (ng/mL)) levels, kidney function parameters (serum creatinine (mg/dL)), and the lipid profile.

Echocardiographic parameters and laboratory tests were evaluated twice; at the beginning of the study and after 12 weeks of follow-up.

### 4.4. Statistical Analysis

The sample size calculation was performed using G.power 3.1.9.2 (Universitat Kiel, Kiel, Germany). The sample size was calculated based on the following considerations: 0.05 α error and 95% power of the study to demonstrate a 9% decrease in PASP in the test group compared to the control group (mean 26.8 and SD 3.2 based upon a previous study) [36]. Six cases were added to overcome dropout. Therefore, 100 patients were allocated in this study. Statistical analyses were carried out using SPSS 26 (IBM Inc., Chicago, IL, USA). Histograms and Shapiro–Wilk tests were used to determine the normality of the data distribution. Student’s *t*-tests were used to compare parametric quantitative variables between groups based on mean ± standard deviation (SD). The median with interquartile range (IQR) of non-parametric quantitative variables was calculated, and the Mann–Whitney U test was used to compare groups, as well as Wilcoxon signed-rank tests to evaluate changes within the same group over time. The chi-square test or Fisher’s exact test was used to compare categorical variables presented as frequencies and percentages. Statistics were considered significant when a two-tailed *p* value < 0.05 was applied.

## 5. Conclusions

In patients with heart failure and PH due to left heart disease, the addition of sildenafil to dapagliflozin did not demonstrate a statistically significant additional clinical or hemodynamic benefit compared to dapagliflozin alone after 12 weeks of treatment. Dapagliflozin remains an effective and well-tolerated option for this population. Further research is required to determine which patients may obtain a therapeutic advantage from combination therapy.

## Figures and Tables

**Table 1:** Baseline data of the studied groups.

		Test Group (*n* = 45)	Control Group (*n* = 48)	Total (*n* = 93)	*p* Value	MD or RR (95%CI)
Demographic Data and Medical History						
Age (years)		51 (46–56)	54.5 (45–62.75)	54 (45–60)	0.287	3 (−2: 7)
Sex	Male	30 (66.67%)	29 (60.42%)	59 (63.44%)	0.531	1.1 (0.81:1.5)
	Female	15 (33.33%)	19 (39.58%)	34 (36.56%)		
Diabetes mellitus		3 (6.67%)	4 (8.33%)	7 (7.53%)	1	0.87 (0.36–2.12)
PCI		3 (6.67%)	6 (12.5%)	9 (9.68%)	0.487	0.67 (0.26–1.7)
CKD		0 (0%)	2 (4.17%)	2 (2.15%)	0.495	---
HCV		1 (2.22%)	0 (0%)	1 (1.08%)	0.483	---
AF		7 (15.56%)	4 (8.33%)	11 (11.82%)	0.345	1.37 (0.83–2.27)
Smoking		5 (11.1%)	4 (8.33%)	9 (9.6%)	0.734	1.17 (0.62–2.18)
New York Heart Association (NYHA)						
NYHA class II		15 (33.33%)	17 (35.41%)	32 (34.4%)	1	0.94 (0.54:1.65)
NYHA class III		30 (66.6%)	31 (64.58%)	61 (65.59%)		
Kidney function tests						
Creatinine (mg/dL)		1 (0.9–1.25)	1.05 (0.8–1.26)	1 (0.9–1.25)	0.379	−0.10 (−0.20–0.10)
Urea (mg/dL)		36 (25.5–53)	35.2 (26.5–44)	36 (25.5–48.7)	0.294	−3.74 (−11–3)
Liver function tests						
ALT (U/L)		24 (15–31)	21.85 (16.53 −32.48)	23 (16–31.7)	0.761	0.7 (−3.6–4.2)
AST (U/L)		26 (24.8–31)	25.95 (20–30)	26 (22–30)	0.310	−1.45 (−4.7–1.8)
Lipid profile						
Cholesterol (mg/dL)		160 (156–200)	158 (154–191.25)	160 (154–192)	0.387	−2 (−14.8–6)
HDL (mg/dL)		46 (42–50)	49 (41–54)	49 (42–54)	0.616	0 (−1–5)
LDL (mg/dL)		92.6 (76–119)	86 (60–122)	91 (63.6–122)	0.352	−5.1 (−20.2–9.2)
Triglyceride (mg/dL)		134 (118.52–177)	134 (91.5–178.05)	134 (108–178)	0.630	−2 (−30.8–16.26)
Cardiac enzymes						
Troponin (ng/mL)		0.1 (0.1–0.11)	0.1 (0.1–0.12)	0.1 (0.1–0.14)	0.625	0 (−0.01–0)
Echocardiographic findings						
EF (%)		30 (26–40)	33.5 (28.5–40)	30 (27–40)	0.362	1 (−2–5)
PASP (mmHg)		50 (45–55)	50 (45–50)	50 (45–53)	0.080	−2 (−5–0)
Treatment						
ACEi or ARB		41 (91.6%)	45 (93.75%)	86 (92.47%)	0.708	0.83 (0.423–1.64)
Beta-blocker		43 (95.5%)	46 (95.83%)	89 (95.69%)	1	0.97 (0.35–2.64)
Aldosterone antagonist		38 (84.44%)	40 (83.33%)	78 (83.87%)	0.884	1.01 (0.85:1.21)
Loop diuretic		39 (86.66%)	38 (79.16%)	77 (82.79%)	0.338	1.09 (0.91:1.32)
Thiazide diuretic		1 (2.22%)	2 (4.16%)	3 (3.22%)	1	1.47 (0.29–7.37)
Statins		15 (33.33%)	17 (35.42%)	32 (34.41%)	0.833	0.94 (0.54:1.65)

Data were presented as median (IQR) or as frequency and percentage (%). MD: median difference, RR: relative risk. PCI: Percutaneous coronary intervention, CKD: Chronic kidney disease, HCV: Hepatitis C virus, AF: Atrial fibrillation, ALT: Alanine aminotransferase, AST: Aspartate aminotransferase. HDL: High density lipoprotein, LDL: Low density lipoprotein. EF: ejection fraction, PASP: pulmonary artery systolic pressure, ACEi: angiotensin-converting enzyme inhibitor, ARB: angiotensin II receptor blocker.

**Table 2:** Change in cardiac enzymes, kidney function parameters, and lipid profile of the studied groups.

			Test Group (*n* = 45)	Control Group (*n* = 48)	*p* Value	MD (95% CI)
Lipid profile	Change in cholesterol (mg/dL)	Median	−31	−6	0.192	12.06 (−5–30.2)
		IQR	−68–3.2	−35.3–2		
	Change in HDL (mg/dL)	Median	2	1.5	0.152	−2 (−5–0.8)
		IQR	−1–10	−3–5		
	Change in LDL (mg/dL)	Median	−30.5	−10.75	0.110	12.4 (−2.5–29.9)
		IQR	−83.4–−2.3	−38.95–−0.95		
	Change in triglyceride (mg/dL)	Median	3	−3.2	0.598	−4.5 (−20.5–14.5)
		IQR	−24–18	−33.5–14.6		
Cardiac enzymes	Change in troponin (ng/mL)	Median	0	0	0.574	0 (0–0.01)
		IQR	−0.01–0.01	−0.01–0.01		
Kidney function	Change in creatinine (mg/dL)	Median	0	0	0.923	0 (−0.14–0.2)
		IQR	−0.1–0.1	−0.3–0.4		

Data were presented as median (IQR). HDL: High density lipoprotein, LDL: Low density lipoprotein, MD: Median difference.

**Table 3:** Safety outcomes.

Causes of Hospitalization	Test Group (*n* = 45)	Control Group (*n* = 48)	*p* Value	RR (95%CI)
Decompensated heart failure due to AF	1 (2.22%)	1 (2.08)	0.524	----
Heart failure and ACS	2 (4.44%)	1 (2.08%)		
DCM and syncopal attack	0 (0%)	1 (2.08%)		
Stroke	0 (0%)	1 (2.08%)		

AF: Atrial fibrillation, ACS: Acute coronary syndrome, DCM: Dilated cardiomyopathy, RR: relative risk.

## Data Availability

The data can not be made public due to privacy of patients. The data will be available upon request from the corresponding author.

## Abbreviations

The following abbreviations are used in this manuscript:

HF	Heart failure
EF	Ejection fraction
PH	Pulmonary Arterial Hypertension
LVEF	Left ventricle ejection fraction
HFrEF	Heart failure with reduced ejection fraction
HFpEF	Heart failure with preserved ejection fraction
HfmrEF	Heart failure with mildly reduced ejection fractions
PH-LHD	PH resulting from left-sided heart disease
SGLT2I	Sodium–glucose cotransporter 2 inhibitors
PDE-5I	selective Phosphodiesterase Type 5 inhibitor
GDMT	Guideline-Directed Medical Therapy
NYHA	New York Heart Association
FMD	flow-mediated dilatation
6MWT	6 min walk test
AF	atrial fibrillation
AEs	adverse events
(ipcPH)	isolated pre-capillary pulmonary hypertension
Cpc-PH	pre- and post-capillary pulmonary hypertension
DAPA	dapagliflozin
ESC	European Society of Cardiology
cAMP	increasing cyclic adenosine monophosphate
